# Whole-Genome Shotgun Sequence of Drug-Resistant Staphylococcus aureus Strain SA9, Isolated from a Slaughterhouse Chicken Carcass in South Africa

**DOI:** 10.1128/MRA.00489-19

**Published:** 2019-06-13

**Authors:** Daniel G. Amoako, Anou M. Somboro, Akebe Luther King Abia, Mushal Allam, Arshad Ismail, Linda A. Bester, Sabiha Y. Essack

**Affiliations:** a Infection Genomics and Applied Bioinformatics Division, Antimicrobial Research Unit, College of Health Sciences, University of KwaZulu-Natal, Durban, South Africa; b Antimicrobial Research Unit, College of Health Sciences, University of KwaZulu-Natal, Durban, South Africa; c Biomedical Resource Unit, School of Laboratory Medicine and Medical Sciences, College of Health Sciences, University of KwaZulu-Natal, Durban, South Africa; d Sequencing Core Facility, National Institute for Communicable Diseases, National Health Laboratory Service, Johannesburg, South Africa; University of Rochester School of Medicine and Dentistry

## Abstract

Here, we describe the genomic sequence of a drug-resistant Staphylococcus aureus sequence type 239 (ST239) strain, SA9, isolated from a slaughterhouse chicken carcass in South Africa, including information about its antibiotic resistome, virulome, efflux genes, clonal lineage, and mobilome. This genomic information offers vital insights for the control of drug-resistant S. aureus.

## ANNOUNCEMENT

Staphylococcus aureus is an important opportunistic pathogen of humans and animals and a leading global cause of foodborne disease (1). Poultry as a potential reservoir for the zoonotic transmission of drug-resistant S. aureus to humans has been well documented (2). Despite the apparent threat, the genomics of antibiotic-resistant S. aureus in food animals appear to be neglected in Africa (3, 4). Toward this end, S. aureus SA9, isolated from a slaughterhouse chicken carcass in South Africa, was subjected to whole-genome sequencing (WGS). Antimicrobial susceptibility testing utilizing the broth microdilution method revealed its multidrug-resistant profile.

Strain SA9 was isolated and confirmed using HiCrome Aureus agar base (HiMedia Laboratories, Mumbai, India) and an API Staph kit (bioMérieux, Marcy-l’Etoile, France), respectively. It was streaked onto Mueller-Hinton agar (MHA) plates and incubated at 37°C for 24 h. Following incubation, genomic DNA was extracted from 1 CFU of a visibly pure culture of the isolate using the GenElute bacterial genomic DNA kit (Sigma-Aldrich, St. Louis, MO, USA), quantiﬁed with a NanoDrop 8000 spectrophotometer (Thermo Scientific, Waltham, MA). A paired-end (2 × 300-bp) library was prepared using an Illumina Nextera XT DNA sample preparation kit and sequenced on a MiSeq machine (Illumina, USA). The generated sequenced reads (1,448,958 reads) were quality assessed and trimmed using the next-generation sequencing (NGS) core tools in the CLC Genomics Workbench version 11.0.1 (CLC bio, Qiagen, Aarhus, Denmark). Default parameters were used for all software unless otherwise specified. The genome was *de novo* assembled using SPAdes version 3.11 (5), and 477 contigs (99× coverage) were obtained, the longest being 79,480 bp, with an *N*_50_ value of 35,831 bp. The CheckM tool (6), using lineage-specific marker sets from other genetically well-characterized, closely related S. aureus strains, was used to verify that the sequence reads were not from mixed species. The NCBI Prokaryotic Genome Annotation Pipeline (PGAP; version 4.3) (7) and Rapid Annotations using Subsystems Technology (RAST) server (version 2.0) (8) were used for annotation. The genome features were as follows: genome size, 3,055,359 bp; GC content, 33.50%; total number of genes, 3,565; total number of coding sequences (CDS), 3,506; number of coding genes, 3,332; number of RNA genes, 59; number of rRNAs, 13; and number of tRNAs, 42.

The molecular typing of strain SA9 was performed *in silico* using the WGS data online platform tools MLST 2.0 (9), *spa*Typer 1.0 (10), and eBURST v3 (11). The multilocus sequence type (ST), *spa* type, and clonal complex (CC) were ST239, t037, and CC8, respectively. PlasmidFinder v1.3 (12) identified the *rep20* plasmid replicon located on contig 178 (GenBank accession no. RQTC01000178). The antibiotic resistome included genes for aminoglycosides [*aac(6')-aph(2''), ant(6)-Ia, aac(3)-II, spc*], beta-lactam (*blaZ*), macrolide (*ermA*), tetracycline (*tetM*), and trimethoprim (*dfrG*) (according to ResFinder through the GoSeqIt tools Web platform) (13). Efflux genes (*qacB*, *mgrA, mepR, arlS*, and *mepA*) associated with the extrusion of antibiotics and acridine dyes were found (14). The PHAge Search Tool Enhanced Release (PHASTER) (15) detected one intact phage (*Staphy_SA13_NC_021863[18]*) with 100% identity. The VirulenceFinder database (16) determined the following virulence factors: hemolysins (*hlb*, *hlgA*, *hlgB*, and *hlgC*), leukotoxins (*lukD* and *lukE*), staphylokinase (*sak*), and enterotoxins (*sea*, *sek*, *sep*, and *seq*) in SA9, allowing it to invade the immune system, adhere to surfaces, colonize, and cause harmful toxic effects to the host (17). To the best of our knowledge, this is the first report of a human-associated clone (ST239) in poultry in South Africa, affirming the recent blurring epidemiology of emerging clones (18). This genomic information will offer significant insights for the control of drug-resistant S. aureus ST239.

### Data availability.

This whole-genome shotgun project has been deposited in DDBJ/ENA/GenBank under the accession no. RQTC00000000. The version described here is the first version, RQTC01000000. The raw reads have been submitted to the SRA under accession no. SRR8583490.
